# Quality Assessment and Host Preference of *Telenomus podisi* (Hymenoptera: Scelionidae) for Fresh and Cryopreserved *Euschistus heros* (Hemiptera: Pentatomidae) Eggs

**DOI:** 10.3390/insects16010086

**Published:** 2025-01-16

**Authors:** Gabryele Silva Ramos, Rafael Hayashida, Pedro Hiroshi Passos Ikuno, Vanessa Rafaela de Carvalho, William Wyatt Hoback, Regiane Cristina de Oliveira

**Affiliations:** 1 Crop Protection Department, Campus of Botucatu, São Paulo State University, Botucatu 18610-307, SP, Brazil; gabryele.sr@gmail.com (G.S.R.); pedro.ikuno@unesp.br (P.H.P.I.); regiane.cristina-oliveira@unesp.br (R.C.d.O.); 2 Department of Entomology and Plant Pathology, Oklahoma State University, Stillwater, OK 74078, USA; whoback@okstate.edu; 3 Department of Functional Genomics & Vector Microbiology, São Paulo State University, Botucatu 18610-307, SP, Brazil; vanessa.carvalho@unesp.br

**Keywords:** pest management, Hemiptera IPM, mass rearing, egg parasitoid, Scelionidae, quality assessment

## Abstract

The development of the mass rearing technique for the egg parasitoid *Telenomus podisi* has been under study for about 20 years, with increasing attention on the development of quality control. Here, we evaluated the behavior, biological parameters, morphometrics and presence of endosymbionts of *T. podisi* produced in cryopreserved eggs compared to those produced in traditional fresh stink bug eggs. Parasitoids reared from cryopreserved eggs showed similar parasitism and emergence rates, sex ratios, longevity, morphometrics, and proportions of flyers compared to those originating from fresh eggs. Slight differences, including an increase in egg-to-adult development time and differences in the presence of endosymbionts, were observed. Despite these differences, we conclude that the use of cryopreserved eggs is suitable for *T. podisi* mass rearing, allowing more options for timed inundative parasitoid releases for biological control.

## Simple Summary

The neotropical brown stink bug is one of the most important pests of soybean, with management frequently relying on the use of chemical insecticides. However, this approach causes environmental impacts, human health risks, and biodiversity loss, while also causing insecticide resistance to build. Alternatively, biological control with mass releases of parasitoids can also be used to manage this pest. For this, reliable and timely parasitoid mass production in bio-factories is required. In this study, we evaluated the quality of parasitoids produced using frozen host eggs, a promising technique that can improve parasitoid mass production. Parasitoids reared from frozen eggs showed similar quality compared to those originating from fresh eggs, which are usually used in bio factories. This indicates that frozen eggs are a suitable tool for parasitoid mass rearing, providing more options for timed releases in soybean fields.

## 1. Introduction

Soybean, *Glycine max* (L.) Merril, is one of the main commodity crops cultivated globally, because of its high yield potential and importance as a source of protein and vegetable oil [1,2]. In the field, soybean crops are subject to attack from various insect pests including caterpillars, leaf beetles, and aphids. Recently, stinkbugs have become the most important pest group of soybean in the Neotropics, which can cause serious injury to plants, leading to substantial economic losses [3]. Recent surveys indicate that the most abundant and important stinkbug pest species in the neotropics is the Neotropical brown stink bug, *Euschistus heros* (Fabricius, 1794) (Hemiptera: Pentatomidae) [4].

Currently, the main approach for stinkbug management in soybean is through the application of chemical insecticides [5,6]. However, this approach can have some undesirable effects, including environmental impacts, the emergence of pest resistance [7] and the reduction in natural biocontrol agents and pollinators [8,9]. In South America, an alternative to chemical insecticides is the use of augmentative biological control (ABC) through mass releases of egg parasitoids [10]. Such biological control agents can reduce pest populations to levels below the economic injury threshold [11,12]. The egg parasitoid *Telenomus podisi* Ashmead, 1893 (Hymenoptera: Scelionidae), is an important and efficient natural enemy that can be adopted in ABC for stinkbug management in soybean fields, with a single female capable of parasitizing over 100 *E. heros* eggs [13]

Although *T. podisi* is currently produced on a large scale in bio-factories using *E. heros* eggs as natural hosts, many aspects of its mass production need further optimization [14]. In this context, cryopreservation of eggs at low temperatures (typically in liquid nitrogen at −196 °C) has been integrated into the *T. podisi* mass rearing techniques. The adoption of cryopreserved *E. heros* eggs is important in the context of ABC programs using *T. podisi*, which can be produced in large quantities and stored for long periods. This allows synchronization with the periods of high demand for parasitoids in soybean fields, without significantly compromising the physical and chemical characteristics of the host eggs [14,15].

Several studies on cryopreserved *E. heros* eggs have shown promising results with minimal impact on the development and biology of *T. podisi* [13,16,17,18]. However, the parasitoid’s morphological and behavioral aspects, such as host preference for fresh versus cryopreserved *E. heros* eggs, the effects of cryopreservation on flight capacity, and the impact on key endosymbiont microorganisms, have not been previously investigated.

Therefore, this study aimed to compare *T. podisi* produced from fresh or cryopreserved *E. heros* eggs, based on their biological and morphometric parameters and flight behavior. In addition, we determined the effect on presence of endosymbiont groups found in these parasitoids that benefit survival and fecundity.

## 2. Materials and Methods

*Euschistus heros* colonies were established from, approximately, 100 adults collected in soybean fields at the Edgardia Experimental Farm (22°49′26″ S, 48°25′07″ W). Both insect colonies and bioassays for the cryopreserved versus fresh eggs (matrix preference test), in addition to offspring morphometric and flight tests, were conducted in the laboratory of the Integrated Pest Management Research Group in Agriculture (AGRIMIP), and the endosymbiont molecular analyses were conducted in the Molecular Biology Laboratory. Both laboratories are located in the Department of Plant Protection at the Faculty of Agricultural Sciences, São Paulo State University, FCA/UNESP, Botucatu Campus, Brazil.

### 2.1. Insects Colonies

*Euschistus heros* colonies were kept under controlled environmental conditions of 70 ± 5% humidity, at a temperature of 25 ± 2 °C, and with a 14/10 h photoperiod (L/D), following the procedures described by Borges et al. [19] and Silva et al. [20]. Adults and third instar nymphs of *E. heros* were kept in plastic cages (4.5 L) lined with filter paper. The insects were fed a natural diet consisting of dry peanut seeds (*Arachis hypogaea* L.), fresh green bean pods (*Phaseolus vulgaris* L.), and privet fruits (*Ligustrum lucidum* L.). A plastic plate (Ø 60 mm) containing cotton soaked in distilled water was placed in each cage as a water supply.

Approximately 100 couples were placed in the *E. heros* adult cages with four pieces of raw cotton fabric (10 × 5 cm) provided for oviposition. The cages were cleaned, food was replaced, and egg masses were collected three times a week. After each collection, the egg masses were transferred to acrylic boxes (11 × 11 × 3.5 cm) lined with filter paper moistened with sterile distilled water (Gerbox^®^). After hatching, second instar nymphs were transferred to new cages identical to adult cages. The laboratory-reared insects were then used for experiments and colony maintenance.

The *T. podisi* colony was maintained on eggs of *E. heros* [21,22]. Twice a week, about 2000 host eggs were glued to pieces of cardboard (2 × 8 cm) and introduced into cages (3 L) containing about 300 *T. podisi* females, sealed with plastic film and elastic bands, and provided with small drops of honey for parasitoid feeding. Parasitism was allowed for 24 h, and then each host eggs’ cardboard was individualized and maintained in new cages until parasitoid emergence.

### 2.2. Bioassays

#### 2.2.1. *Telenomus podisi* Host Preference Test

A two-choice preference test for fresh or cryopreserved *E. heros* eggs in liquid nitrogen (−196 °C) was performed in arenas adapted from the methodology described by Thuler et al. [23] (Figure S1). The arenas were made using 50 mL Falcon™ tubes (Fisher Scientific, Waltham, MA, USA) with four 4 mL acrylic tubes (Duran tubes), arranged equidistantly. Twenty arenas were randomly arranged, with each arena receiving four 4 × 1 cm paper cards with 40 *E. heros* eggs and with two paper cards of each treatment in each Duran tube, and each arena was considered as one replication. The treatments consisted of fresh eggs up to 24 h old, and cryopreserved eggs stored for 30 days at −196 °C in liquid nitrogen. The cryopreserved eggs were thawed in aluminum foil by immersion in water at 35 °C for five seconds and dried before being glued to the paper cards.

Four *T. podisi* females from fresh *E. heros* eggs were released through a central upper hole and were able choose between the two types of eggs for parasitism. After 24 h, the cards were removed from the arenas, placed in individual Duran tubes, and maintained in a biochemical oxygen demand (BOD) incubator under the same conditions described for *T. podisi* colony rearing. After 10 days of development, the daily emergence of parasitoids was counted for 10 days to access egg-to-adult development time, and sex ratio [number of females/(number of males + number of females)], egg-to-adult development time, parasitism rate (calculated by number of parasitized eggs divided by the total number of offered eggs) and emergence rate (calculated by dividing the number of emerged adults by the total number of parasitized eggs) were evaluated. Fifteen females from each treatment were individualized in Duran tubes and fed daily with honey drops to access the survival.

Simultaneously, another set of the test was conducted using four females originating from cryopreserved eggs following the same protocol. From these two independent experiments, we obtained four different treatments: *T. podisi* from fresh eggs parasitizing fresh eggs (FF), *T. podisi* from fresh eggs parasitizing cryopreserved eggs (FC), *T. podisi* from cryopreserved eggs parasitizing fresh eggs (CF), and *T. podisi* from cryopreserved eggs parasitizing cryopreserved eggs (CC). These treatments were compared in the subsequent analysis. Samples (N = 50) from each treatment were stored in Chelex fixative for endosymbiont identification tests [24] and other samples (N = 15 for each treatment) were stored in 70% alcohol for the study of morphometric characters.

Additionally, a no-choice test was conducted. In this experiment, we evaluated the oviposition capacity of *T. podisi* females originating from fresh or cryopreserved eggs to parasitize fresh and cryopreserved eggs as hosts. Each female was placed into a flat-bottom glass tube (Ø 20 mm × 80 mm height), fed with a honey drop and allowed to oviposit over a 24 h period. We tested 20 replications for each treatment, with a card containing 40 eggs in each. After the oviposition period, the females were removed, and the cards were individually stored and analyzed under the same conditions as those described for the choice test.

#### 2.2.2. Identification of Endosymbionts

Pools of fifty adults of *T. podisi* collected from each treatment (FF, FC, CF, and CC), plus 50 *E. heros* fresh eggs up to 24 h old and 50 cryopreserved *E. heros* eggs (FE and CE, respectively) were used for the identification of endosymbionts. Egg samples used for the experiments were collected from different ovipositions of about 100 couples kept under controlled conditions in the laboratory, ensuring the representativeness of the batches. From each pool of 50 individuals or 50 eggs (one replicate), DNA extraction was performed by macerating the material in a polypropylene tube (Eppendorf, Hamburg, Germany) with the addition of 50 µL of 10% Chelex 100^®^ solution and 5 µL of protease K [25]. The mixture was incubated for 20 s at 95 °C to disrupt the cell membranes and tissues. Subsequently, the samples were subjected to duplicate PCR amplification with primers specific for the endosymbionts listed in Table S1 (Supplementary Materials). The list of primers, their references, and the cycle and temperature conditions used for each endosymbiont detection are available in Table S1 [26,27,28,29,30,31,32,33,34].

The PCR samples obtained for each treatment in which a positive band for the tested endosymbiont was present were purified and quantified according to the technological purification procedure using magnetic beads on a magnetic plate. The procedure consisted of the homogeneous addition of 40 µL of BEAD extractor to 20 µL of the PCR solution. The solution was then placed on a magnetic plate for three minutes to separate the sample DNA from the solution. Subsequently, washes with 70% alcohol were performed, and the DNA was eluted in nuclease-free water. The DNA concentration of the sample was measured using a NanoDrop^®^ 2000 Spectrophotometer (Thermo Scientific, Carlsbad, CA, USA). When the DNA concentration in nanograms/µL was sufficient for each endosymbiont, these samples were sent for automatic Sanger DNA sequencing (Model ABI 3500—Applied Biosystems) at the Biotechnology Institute of UNESP Rubião Campus (IBTEC).

Endosymbiont presence was confirmed through comparisons with sequences obtained from the sequencing of the 16S (and 23S for *Arsenophonus* sp. only) rRNA regions and endosymbiont sequences deposited in NCBI’s GenBank. Comparisons were made by multiple alignments generated using the BLAST algorithm (https://blast.ncbi.nlm.nih.gov/Blast.cgi [accessed on 15 May 2020]), with similarity criteria greater than 97% to confirm the identity of the detected symbionts.

#### 2.2.3. Morphometry

Samples of 15 females and 15 males from each treatment (FF, FC, CF, and CC) were stored in 70% alcohol for the study of morphometric characters on the right side of the body (wing width and length, tibia size, and body length) using a stereomicroscope (Leica Application Suite-Version 1.6.0) (Supplementary Figure S2). Each insect was considered a replication (N = 15 for females and N = 15 for males).

#### 2.2.4. Flight Capacity of Offspring

The flight tests of *T. podisi* were conducted in PVC pipe cages following the IOBC model adapted from Prezotti [35]. The inner part of the PVC pipe was lined with black cardboard, and the bottom was sealed with Styrofoam discs covered with black paper and plastic film (Figure S2). To detect the presence of walker parasitoids, a 0.5 cm wide acetate strip covered with entomological glue was fixed 3.5 cm from the base of the cage. The top of the tube was sealed with a Petri dish (Ø 150 mm) coated with entomological glue to capture flying parasitoids. Tests were conducted for the offspring of each treatment (FF, FC, CF, and CC). Couché paper circles (Ø 20 mm) containing parasitized *E. heros* eggs of each treatment were placed in flat-bottom glass tubes (Ø 20 mm × 80 mm height) and fixed at the center of each cage. Twenty cages per treatment were then placed on a bench with direct and constant lighting. After five days, the flyers (trapped in the upper part of the test cage), walkers (trapped on the acetate strip), and immobile parasitoids (remaining at the bottom of the test cage) were counted.

### 2.3. Statistical Analyses

The parameters evaluated in each bioassay (sex ratio, egg–adult development duration, parasitism and emergence rate), as well as the morphometry data, underwent analysis of variance (ANOVA). Before proceeding with the ANOVA, we conducted exploratory data analysis to assess the normality assumptions of the residuals [36] and homogeneity of variances [37]. The means were compared using the Tukey post hoc test at a significance level of α = 0.05. When the data followed a non-parametric distribution, they were subjected to Kruskal–Wallis analysis and the medians were compared using the Dunn test at a significance level of α = 0.05. Longevity data were assessed using the Kaplan–Meier statistical method and compared using the Log-rank test. All analyses and graphics were made using SigmaPlot software version 12.0 (Systat Software, San Jose, CA, USA).

## 3. Results

### 3.1. Host Preference Test

**Figure 1:**
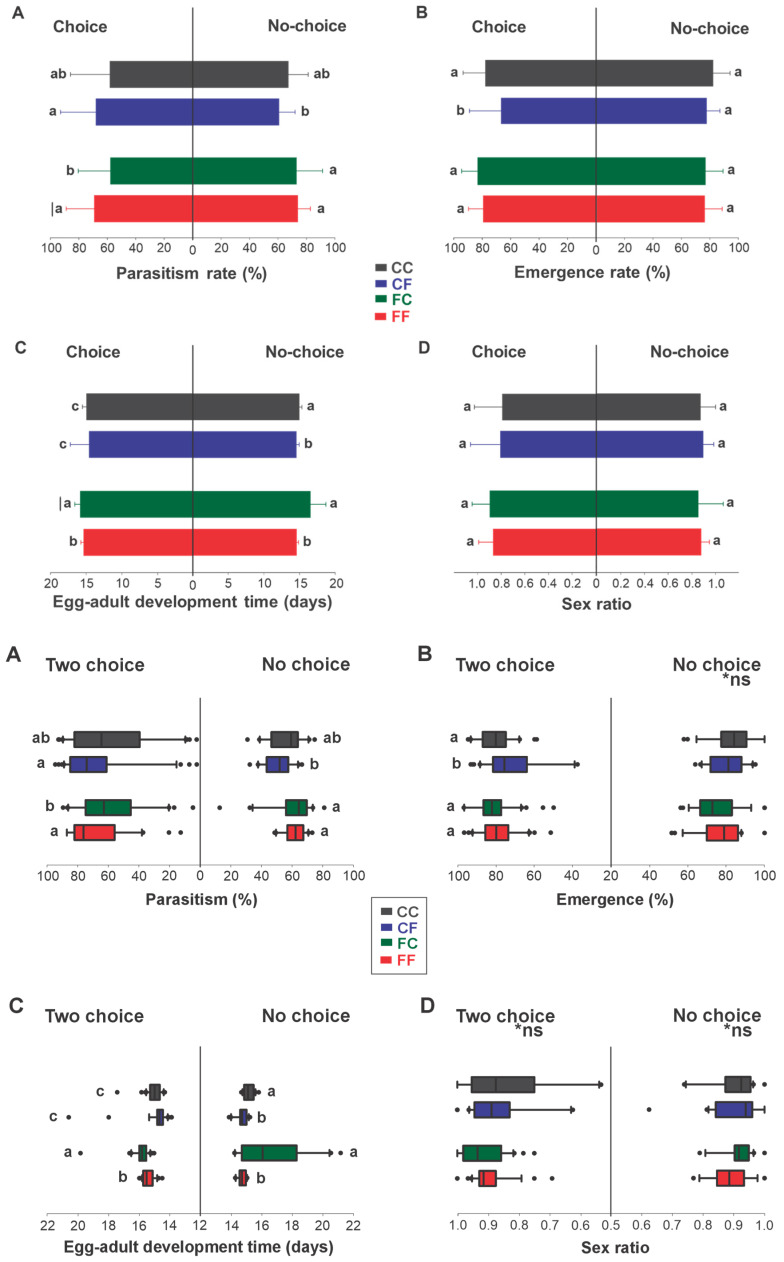
*Telenomus podisi* (Hymenoptera: Scelionidae) biological parameters in different treatments for two-choice (left side) and no-choice (right side) bioassays: Parasitism (**A**) and percent emergence rate (**B**), egg–adult development time in days (**C**) and sex ratio [number of females/(number of males + number of females)] (**D**). Bars (means ± standard error) followed by the same letter do not differ according to Tukey’s test at 5% significance. Box plots (medians ± IQR) followed by the same letter do not differ according to the H test at 5% significance. CC (grey): adults from cryopreserved eggs parasitizing cryopreserved eggs; CF (blue): adults from cryopreserved eggs parasitizing fresh eggs; FC (green): adults from fresh eggs parasitizing cryopreserved eggs; and FF (red): adults from fresh eggs parasitizing fresh eggs. *ns = Non-significant differences.

Minor but significant differences in the parasitism and emergence rates of *T. podisi* were observed in the choice preference test. The highest parasitism rate was observed in females from fresh eggs parasitizing fresh eggs (FF) and females from cryopreserved eggs parasitizing fresh eggs (CF); 76.59 ± 25.24% and 74.46 ± 22.26%, respectively (H = 9.56; df = 3; *p* = 0.023; Figure 1). Parasitism of cryopreserved eggs was approximately 20% lower when given a choice. The emergence rate was significantly lower when females from cryopreserved eggs parasitized fresh eggs (CF), with 75.74 ± 17.45% of adults emerging from the parasitized eggs (H = 6.35; df = 3; *p* = 0.012; Figure 1). When the females were exposed to the no-choice test, the highest parasitism rates were observed in the FF and FC treatments, with 75.00 ± 11.16% and 76.50 ± 14.17%, respectively (H = 14.85; df = 3; *p* = 0.002; Figure 1); however, no differences were observed in the emergence rate.

The egg-to-adult development time in the two-choice bioassays was significantly shorter when matrices came from cryopreserved eggs (CC and CF), taking a median of 14.75 ± 0.56 days and being about 8% faster for the emergence of adults (H = 78.27; df = 3; *p* < 0.001; Figure 1). However, when there was no choice, the treatment with fresh host eggs (CF and FF), regardless of the origin, showed the fastest egg-to-adult development, with a median of 14.68 ± 0.38 days (H = 21.27; df = 3; *p* = 0.0001; Figure 1). No differences in sex ratio were observed among treatments, in either choice or no-choice tests (Figure 1).

**Figure 2:**
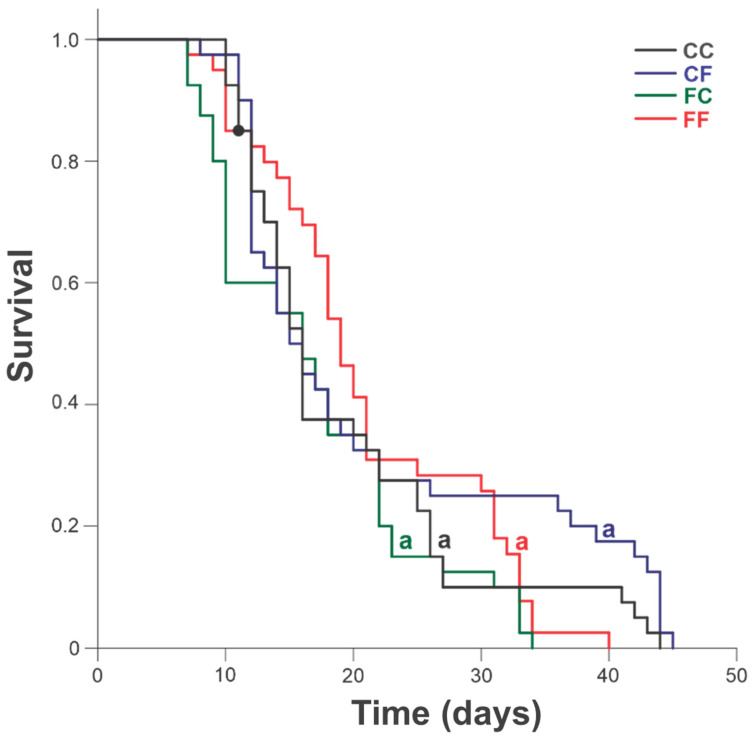
*Telenomus podisi* (Hymenoptera: Scelionidae) female longevity: CC (grey): adults from cryopreserved eggs parasitizing cryopreserved eggs; CF (blue): adults from cryopreserved eggs parasitizing fresh eggs; FC (green): adults from fresh eggs parasitizing cryopreserved eggs; and FF (red): adults from fresh eggs parasitizing fresh eggs. Curves followed by the same letter do not differ according to the Holm–Sidak method at 5% significance (Kaplan–Meier analysis: Log-rank method). Black point indicates missing female.

No differences were observed in the longevity of adults among treatments. The medians ranged from 15.50 ± 12.00 to 19.00 ± 14.50 days for adult survival after emergence (Log-rank analysis = 6.32; *p* = 0.097; Figure 2).

### 3.2. Identification of Endosymbionts

Sequencing by the Sanger method performed during each PCR indicates the presence of *Wolbachia pipientis* (Rickettsiaceae) and *Serratia grimesii* (Enterobacteriaceae) in nearly all the *T. podisi* treatments, except CC, which lacked *S. grimesii*. No tested endosymbionts were found in either fresh or cryopreserved *E. heros* eggs (FE and CE; Table 1).

The results presented in Table 1 reflect the analyses performed on pools of 50 individuals or eggs per treatment. The identification of endosymbionts was based on the amplification and sequencing of specific regions of ribosomal DNA (16S and 23S), followed by comparison with reference sequences in GenBank, ensuring reliable detection.

### 3.3. Morphometry

**Figure 3:**
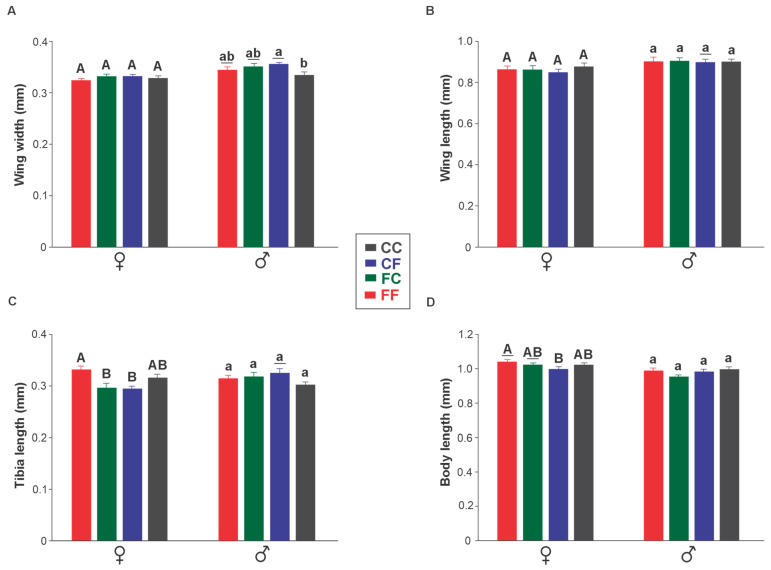
Morphometric parameters of females and males of *Telenomus podisi* (Hymenoptera: Scelionidae). Treatments: FF (red): adults from fresh eggs parasitizing fresh eggs; FC (green): adults from fresh eggs parasitizing cryopreserved eggs; CF (blue): adults from cryopreserved eggs parasitizing fresh eggs; and CC (grey): adults from cryopreserved eggs parasitizing cryopreserved eggs. Wing width (**A**), wing length (**B**) of the right wing, right tibia length (**C**), and body length (**D**). Bars (means ± standard error) followed by the same uppercase letter (comparison among females) and lowercase letter (comparison among males) do not differ according to Tukey’s test at 5% significance. A line below the letter indicates a significantly different mean between males and females within the same treatment.

Significant differences were observed in wing width among males, with the males from CC presenting the smallest wing width, while CF had the largest (F [3, 56] = 3.08; *p* = 0.035). In contrast, no differences were observed in female wings. In general, males presented larger wing width compared to females within the same treatment (F [3, 28] = 16.06; *p* = 0.0004), except in CC, where no significant difference was observed. No differences were observed in wing length among treatments (F [3, 28] = 2.15; *p* = 0.153; Figure 3). Tibia length and body length were significantly higher in females from FF (F [3, 5] = 6.70; *p* < 0.001; and F [3, 56] = 7.42; *p* = 0.011, respectively), while no differences were observed among males.

### 3.4. Flight Capacity of Emmerged Adults

**Figure 4:**
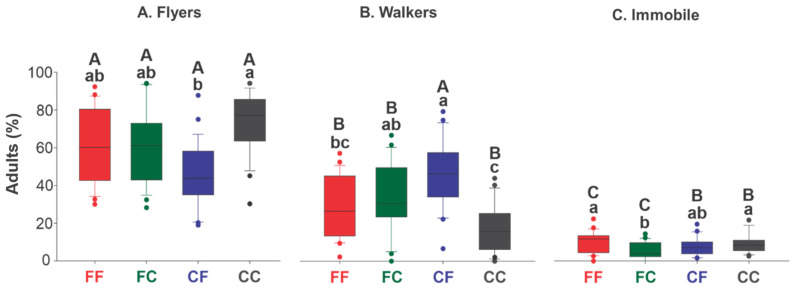
Percentage of flyer (**A**), walker (**B**), and immobile insects (**C**) (%) of *Telenomus podisi* (Hymenoptera: Scelionidae) adults from treatments: FF (red): adults from fresh eggs parasitizing fresh eggs; FC (green): adults from fresh eggs parasitizing cryopreserved eggs; CF (blue): adults from cryopreserved eggs parasitizing fresh eggs; and CC (grey): adults from cryopreserved eggs parasitizing cryopreserved eggs. Box-chart bars followed by the same lowercase letter (comparison among treatments within the same group of flyer, walker, and immobile insects) and uppercase letter (comparison of the same treatment among different groups) do not differ according to the Tukey test at 5% significance. Outliers, represented by circles, are 1.5 times the length of the upper and lower edges of the box, and the second quartile of the boxes represents the median.

The highest proportion of flyers of both sexes was found in CC, with an average of about 80%, while the lowest was found in FC, with an average of about 40% (F [3, 76] = 6.38; *p* < 0.001, Figure 4). Interestingly, the highest proportion of walkers was also found in CF (F [3, 76] = 9.47; *p* < 0.001), and the lowest percentage of immobile insects was found in CF (H = 8.62; df = 3; *p* = 0.035).

When comparing the treatments, CF had an equivalent proportion of flyers and walkers, each greater than 45% (F [3, 57] = 42.45; *p* < 0.001). In contrast, CC had lower proportions of walkers and immobile adults compared with flyers (H = 39.71; df = 3; *p* < 0.001; Figure 4).

## 4. Discussion

Our results corroborate previous studies that support the suitable use of *E. heros* cryopreserved eggs for *T. podisi* mass rearing [13,16,17,18]. Considering the context of ABC programs, where a bio-factory adopts a recurrent use of females that come from cryopreserved eggs parasitizing cryopreserved eggs (CC), our results validate the use of CC and showed that the parasitism rate (%), emergence rate (%), sex ratio, and longevity were equivalent to those originating from fresh eggs parasitizing fresh eggs (FF). Because FF can be considered the treatment most similar to field conditions—where females from fresh eggs parasitize fresh eggs—this result suggests that CC is also suitable for releases in the field.

Similarly, previous studies have reported the suitable use of cryopreserved eggs of *Mythimna sequax* (Lepidoptera: Noctuidae) stored in liquid nitrogen for 90 days for *Trichogramma pretiosum* (Hymenoptera: Trichogrammatidae), with females showing parasitism rates comparable to those reared on fresh eggs [38]. Additionally, ref. [39] reported that *T. pretiosum* can be reared for three generations using the same cryopreserved host without any performance loss. Nevertheless, some important differences were noted. The egg-to-adult development time in the no-choice test was slightly increased in the treatments where females were exposed to cryopreserved eggs (CC and FC). The CC and FC treatments needed at least one more day to complete their development compared to their counterparts with fresh eggs. In the two-choice test, however, the treatments with females that came from cryopreserved eggs (CC and CF) resulted in the shortest egg-to-adult development. Using mathematical models, however, Oliveira et al. [16] observed no significant differences in parasitoid development time for fresh or cryopreserved eggs under different temperatures. Thus, our observed slight differences between the no-choice and two-choice tests and in the scientific literature [16] might be attributed to the origin of the females and conditions under which both experiments were conducted.

Success of any ABC program is dependent on the quality of offspring produced in the bio-factories; thus, flight and performance tests are crucial to ensure that the parasitoids produced from artificial conditions will maintain their foraging behavior consistent with the females found in the fields [35,40,41]. Our results from morphometric and flight capacity tests showed that both treatments using cryopreserved host eggs (CC and FC) produced females with equivalent wing dimensions and proportions of flying insects compared to those originating from a female that came from a fresh egg parasitizing fresh eggs. Thus, in the fields, these females will also maintain their behavior of finding fresh *E. heros* eggs.

Our findings also support that the cryopreserved host eggs present good quality, which did not affect the sex ratio. According to Oliveira et al. [16], the host quality can influence the *T. podisi* sex ratio in two ways: first, it involves the females recognizing the host egg condition and laying different proportions of male and female eggs. Alternatively, after the eggs are laid, host quality allows the developing offspring to determine the sex ratio. In this case, both male and female eggs are laid, but only one sex will survive [42].

The emergence rates of females from cryopreserved eggs parasitizing fresh eggs in choice tests were significantly lower compared to the other treatments, while no difference was observed in the no-choice test. This discrepancy may indicate the behavioral preferences of parasitoid females, as choice tests permit them to exhibit selective oviposition behaviors. Females may choose to parasitize their eggs with greater genetic variability and higher nutritional content on hosts perceived as higher quality, leading to suboptimal parasitism on cryopreserved eggs. In contrast, the no-choice tests do not allow females to present their host preference, potentially contributing to a more deliberate and optimized oviposition on all available hosts. Future studies can investigate the interaction between host quality and female decision-making under diverse experimental conditions to better understand this observation [43,44,45,46].

Additionally, the pooled sample analysis approach allowed efficient detection of endosymbionts, such as *W. pipientis* and *S. grimesii*, ensuring the representativeness of treatments and reflecting possible impacts of egg conditions on the associated microbial community. The presence of endosymbionts might also impact the sex ratio of the progeny produced [43], which can impact the success of the parasitoid production. For example, *Wobachia* sp. can manipulate the parasitoid’s reproductive systems and affect offspring sex ratios [43]. It can limit reproduction by causing reproductive incompatibility, feminization, and parthenogenesis [47]. Our results indicated that *W. pipientis* was fixed in the population of *T. podisi* tested in this work [41] and was not influenced by egg condition. Because *W. pipientis* was not found in either frozen or fresh *E. heros* eggs, the cryopreservation process appears not to affect the presence and transovarian passage of *W. pipientis* in the *T. podisi* parasitism and reproduction process.

In contrast, the presence of *S. grimesii* was conditioned to the transfer of females through fresh eggs, as observed by the lack of this bacteria in the insects from CC. In the eucalyptus snout beetle *Gonipterus platensis* Marelli, 1927 (Coleoptera: Curculionidae), *S. grimesii* is reported to be vertically transmitted to the progeny [48]. However, little is known about the transmission modes and the role of *S. grimesii* in *T. podisi*. More studies are required to evaluate the role of *S. grimesii* in *T. podisi* biology, the mode of transmission of this endosymbiont in laboratory/bio-factory conditions, and the impact of its presence or absence on the parasitoid’s fitness.

## 5. Conclusions

Our findings bolster previous conclusions that cryopreserved *E. heros* eggs are suitable for *T. podisi* mass rearing. Adult biology, behavior, and presence of endosymbionts are equivalent to those reared in fresh eggs, regardless of the egg origin. Despite these results, quality assessment protocols should be conducted periodically to avoid fitness loss in long-term production using cryopreserved eggs, which could include a reduction in parasitism rate, or foraging and flying abilities, as well as changes in the presence of microorganisms [49,50].

## Figures and Tables

**Table 1:** Endosymbiont genera present (+) in *Telenomus podisi* (Hymenoptera: Scelionidae) treatments: FF: *T. podisi* matrix from *Euschistus heros* (Fabricius) (Hemiptera: Pentatomidae) fresh eggs parasitizing fresh eggs; FC: adults from fresh eggs parasitizing cryopreserved eggs; CF: adults from cryopreserved eggs parasitizing fresh eggs; and CC: adults from cryopreserved eggs parasitizing cryopreserved eggs; FE. *E. heros* fresh eggs and CE: *E. heros* cryopreserved eggs. Genera tested: Ars.: *Arsenophonus* sp.; Card.: *Cardinium* sp.; Cars.: *Carsonella* sp.; Ham.: *Hamiltonella* sp.; Nos.: *Nosema* sp.; Reg.: *Regiella* sp.; Ric.: *Rickettsia* sp.; Ser.: *Serratia* sp., Sod.: *Sodalis* sp.; Spi.: *Spiroplasma* sp. and Wol.: *Wolbachia* sp.

Endosymbionts											
	*Ars.*	*Card.*	*Ham.*	*Reg.*	*Ric.*	*Ser.*	*Sod.*	*Spi.*	*Wol.*	*Nos.*	*Cars.*
FF	-	-	-	-	-	+	-	-	+	-	-
FC	-	-	-	-	-	+	-	-	+	-	-
CF	-	-	-	-	-	+	-	-	+	-	-
CC	-	-	-	-	-	-	-	-	+	-	-
FE	-	-	-	-	-	-	-	-	-	-	-
CE	-	-	-	-	-	-	-	-	-	-	-

## Data Availability

The data presented in this study are available on request from the corresponding author.

## Supplementary Materials

The following supporting information can be downloaded at https://www.mdpi.com/article/10.3390/insects16010086/s1: Figure S1—Experimental arenas for the preference tests of *Telenomus podisi* in fresh and cryopreserved eggs of *Euschistus heros*. Arrangement of the arenas during the experiment (A); lateral-top view of each arena (B). Figure S2—Side view of a male *Telenomus podisi* Ashmead (Hymenoptera: Scelionidae) exemplifying how the morphometric study was conducted, measuring the width and length of wings and the size of tibia and body (in red) from photos taken via a stereomicroscope. Figure S3—Schematic illustration of the flight cage model recommended by the IOBC and adapted by Prezotti [35]: Petri dish coated with entomological glue for capturing flyers insects (A); flat-bottom glass tube containing the egg card from which *T. podisi* adults emerged and where immobile insects were observed (B); acetate ring positioned 3.5 cm from the base and coated with entomological glue for collecting walker insects (C). Table S1. Endosymbionts tested in *Telenomus podisi* adults and *Euschistus heros* eggs, primers adopted and references.
