# Supplementary material for: Quality Assessment and Host Preference of Telenomus podisi (Hymenoptera: Scelionidae) for Fresh and Cryopreserved Euschistus heros (Hemiptera: Pentatomidae) Eggs
Source: Insects. 2025 Jan 16;16(1):86. doi: 10.3390/insects16010086 (PMC11766306; doi:10.3390/insects16010086)
Supplement: Supplementary file 1 [file insects-16-00086-s001.zip › insects-3368043-supplementary.pdf]

Table S1. Endosymbionts tested in *Telenomus podisi* Ashmead (Hymenoptera: Platygastridae) adults and *Euschistus heros* (Fabricius) (Hemiptera: Pentatomidae) eggs, primers, amplification information and references.

Endosymbiont	Target Gene	Primer Sequence 5′>3′	bp	Amplification Information	Reference[1]
Arsenophonus	*23S rRNA*	F-CGTTTGATGAATTCATAGTCAAA<br>R-GGTCCTCCAGTTAGTGTTACCCAAC	600	95ºC for 2′, followed by 30 cycles of 95ºC for 30″, 58ºC for 30″, 72ºC for 1′ and final extension at 72ºC for 5′	[1]
Cardinium	*16S rRNA*	F-TACTGTAAGAATAAGCACCGGC<br>R-GTGGATCACTTAACGCTTTCG	900	95ºC for 2′, followed by 30 cycles of 92ºC for 30″, 57ºC for 30″, 72ºC for 30″ and final extension of 72ºC for 5′	[2]
Hamiltonella	*16S rRNA*	F-TGAGTAAAGTCTGGAATCTGG<br>R-AGTTCAAGACCGCAACCTC	700	95ºC for 2′, followed by 30 cycles of 95ºC for 30″, 58ºC for 30″, 72ºC for 1′ and final extension at 72ºC for 5′	[3]
Nosema	*16S rRNA*	F- CACCAGGTTGATTCTGCC<br>R-TTATGATCCTGCTAATGGTTC	222	95ºC for 4′, followed by 45 cycles of 95ºC for 1′, 48ºC for 1′, 72ºC for 1′ and final extension of 72ºC for 4′	[4]
Rickettsia	*16S rRNA*	F-GCTCAGAACGAACGCTATC<br>R-GAAGGAAAGCATCTCTGC	900	95ºC for 2′, followed by 30 cycles of 92ºC for 30″, 58ºC for 30″, 72ºC for 30″ and final extension of 72ºC for 5′	[5]
Serratia	*16S rRNA*	F- CGCAGGCGGTTTGTTAAGTC<br>R- CTTCAAGGGCACAACCTCCA	268	95ºC for 10′, followed by 35 cycles of 95ºC for 1′, 62ºC for 1′, 72ºC for 1′ and final extension of 72ºC for 1′	[6]
Sodalis	*16S rRNA*	F-ACCGCATAACGTCGCAAGACC<br>R-<br>CTTAACCCAACATTTCTCAACACGAG	1000	94ºC for 5′, followed by 30 cycles of 94ºC for 1′, 62ºC for 1′, 72ºC for 2′ and final extension of 72ºC for 5′	[7]
Spiroplasma	*16S rRNA*	F-GCTTAACTCCAGTTCGCC<br>R-CCTGTCTCAATGTTAACCTC	800	94ºC for 5′, followed by 30 cycles of 94ºC for 1′, 52ºC for 1′, 72ºC for 2′ and final extension of 72ºC for 5′	[8]
Wolbachia	*16S rRNA*	F-CGGGGGAAAAATTTATTGCT<br>R-AGCTGTAATACAGAAAGTAAA	700	95ºC for 3′, followed by 30 cycles of 95ºC at 30″, 55ºC at 30″, 72ºC at 30″ and final extension of 72ºC for 5′	[9]

[1]Supplementary References from **Table S1:**

1. Thao, M.L.L.; Baumann, P. Evidence for multiple acquisition of *Arsenophonus* by whitefly species (Sternorrhyncha: Aleyrodidae). *Curr Microbiol* **2004**, *48*, 140–144, doi:10.1007/s00284-003-4157-7.
2. Zchori-Fein, E.; Perlman, S.J. Distribution of the bacterial symbiont *Cardinium* in arthropods. *Mol Ecol* **2004**, *13*, 2009–2016, doi:10.1111/j.1365-294X.2004.02203.x.
3. Zchori-Fein, E.; Brown, J.K. Diversity of prokaryotes associated with *Bemisia tabaci* (Gennadius) (Hemiptera: Aleyrodidae). *Ann Entomol Soc Am* **2002**, *95*, doi:https://doi.org/10.1603/0013-8746.
4. Vossbrinck, C.R.; Baker, M.D.; Didier, E.S.; Debrunner-Vossbrinck, B.A.; Shadduck, J.A. Ribosomal DNA Sequences of Encephalitozoon Hellem and Encephalitozoon Cuniculi: Species Identification and Phylogenetic Construction. *Journal of Eukaryotic Microbiology* **1993**, *40*, 354–362, doi:10.1111/j.1550-7408.1993.tb04928.x.
5. Gottlieb, Y.; Ghanim, M.; Chiel, E.; Gerling, D.; Portnoy, V.; Steinberg, S.; Tzuri, G.; Horowitz, A.R.; Belausov, E.; Mozes-Daube, N.; et al. Identification and localization of a Rickettsia sp. in *Bemisia tabaci* (Homoptera: Aleyrodidae). *Appl Environ Microbiol* **2006**, *72*, 3646–3652, doi:10.1128/AEM.72.5.3646-3652.2006.
6. Zhu, H.; Sun, S.J.; Dang, H.Y. PCR detection of Serratia spp. using primers targeting pfs and luxS genes involved in AI-2-dependent quorum sensing. *Curr Microbiol* **2008**, *57*, doi:10.1007/s00284-008-9197-6.
7. Nováková, E.; Hypša, V. A new Sodalis lineage from bloodsucking fly Craterina melbae (Diptera, Hippoboscoidea) originated independently of the tsetse flies symbiont Sodalis glossinidius. *FEMS Microbiol Lett* **2007**, *269*, 131–135, doi:10.1111/j.1574-6968.2006.00620.x.
8. Montenegro, H.; Solferini, V.N.; Klaczko, L.B.; Hurst, G.D.D. Male-killing *Spiroplasma* naturally infecting *Drosophila melanogaster*. *Insect Mol Biol* **2005**, *14*, 281–287, doi:10.1111/j.1365-2583.2005.00558.x.
9. Heddi, A.; Grenier, A.M.; Khatchadourian, C.; Charles, H.; Nardon, P. Four intracellular genomes direct weevil biology: Nuclear, mitochondrial, principal endosymbiont, and Wolbachia. *Proc Natl Acad Sci U S A* **1999**, *96*, 6814–6819, doi:10.1073/pnas.96.12.6814.
10. Prezotti, L.; Parra, J.R.R.; Vencovsky, R.; Dias, C.T.D.S.; Cruz, I.; Chagas, M.C.M. Teste de vôo como critério de avaliação da qualidade de *Trichogramma pretiosum* Riley (Hymenoptera: Trichogrammatidae): Adaptação de metodologia. *Neotrop Entomol* **2002**, *31*, 411–417, doi:10.1590/s1519-566x2002000300010.

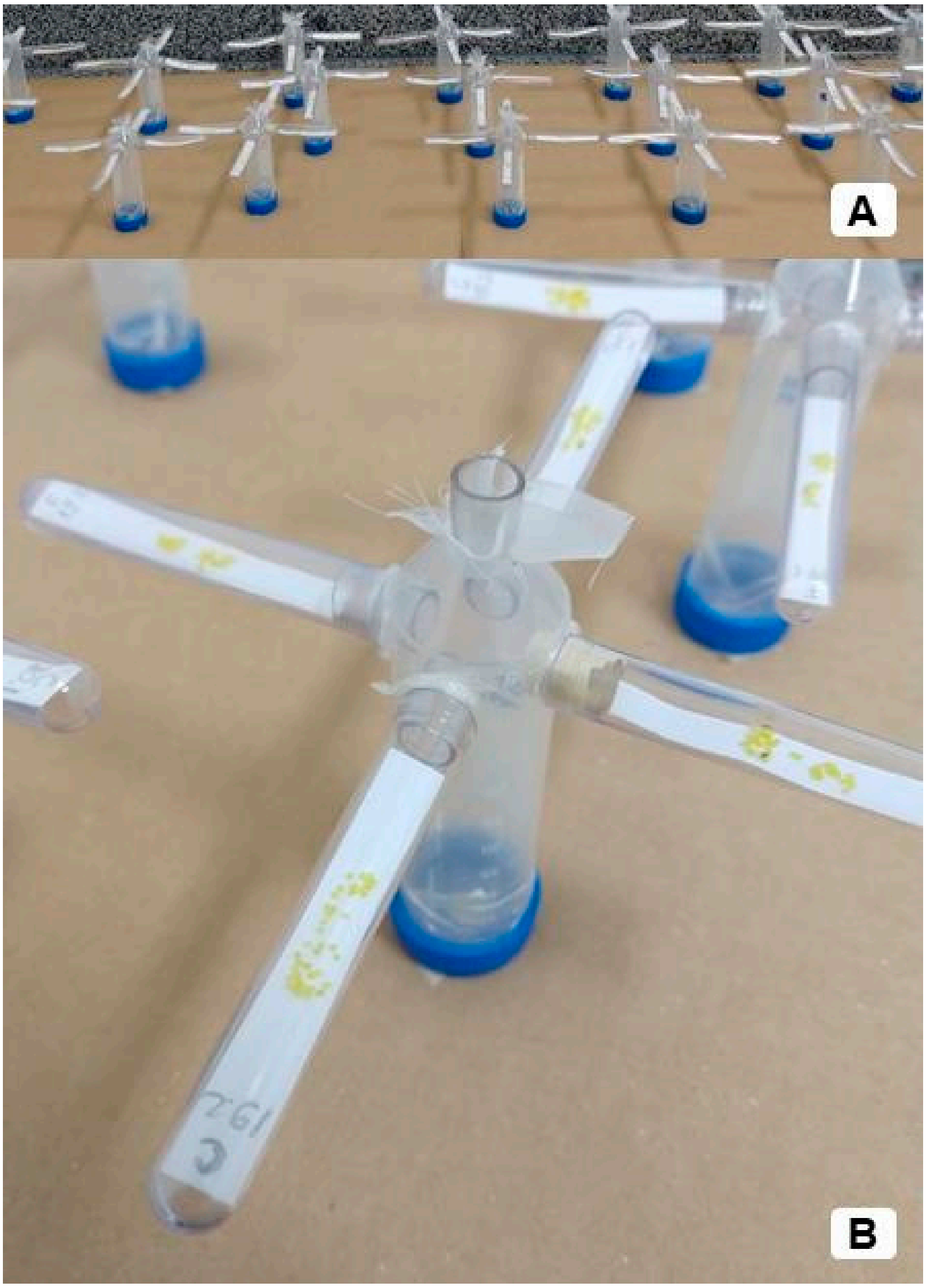

Figure S1 - Experimental arenas for the preference tests of *Telenomus podisi* in fresh and cryopreserved eggs of *Euschistus heros*. Arrangement of the arenas during the experiment (A); lateral-top view of each arena (B).

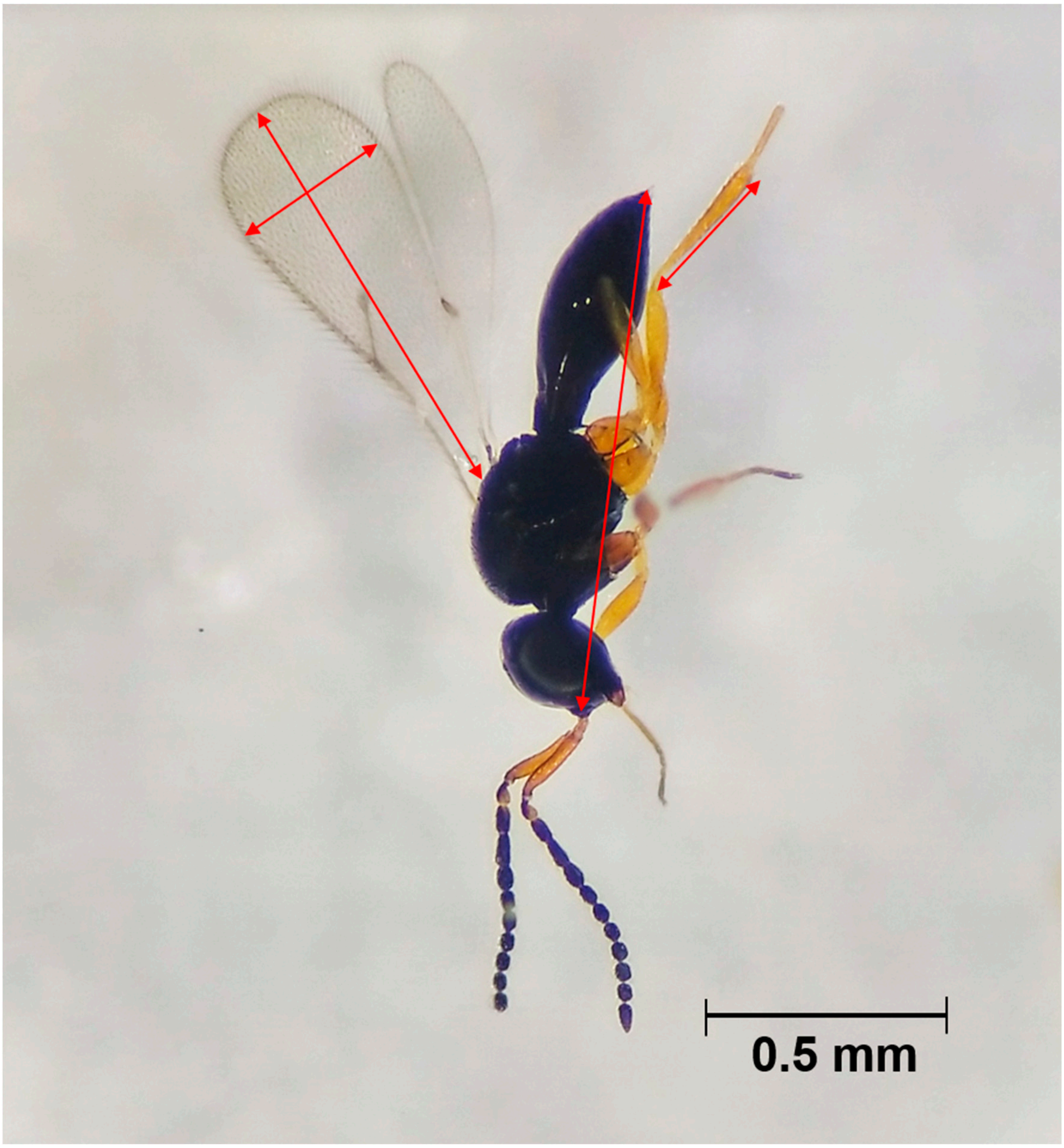

Figure S2 - Side view of a male *Telenomus podisi* Ashmead (Hymenoptera: Scelionidae) showing how the morphometric study was conducted, measuring the width and length of wings and the length of tibia and body (in red) from images taken via stereomicroscope.

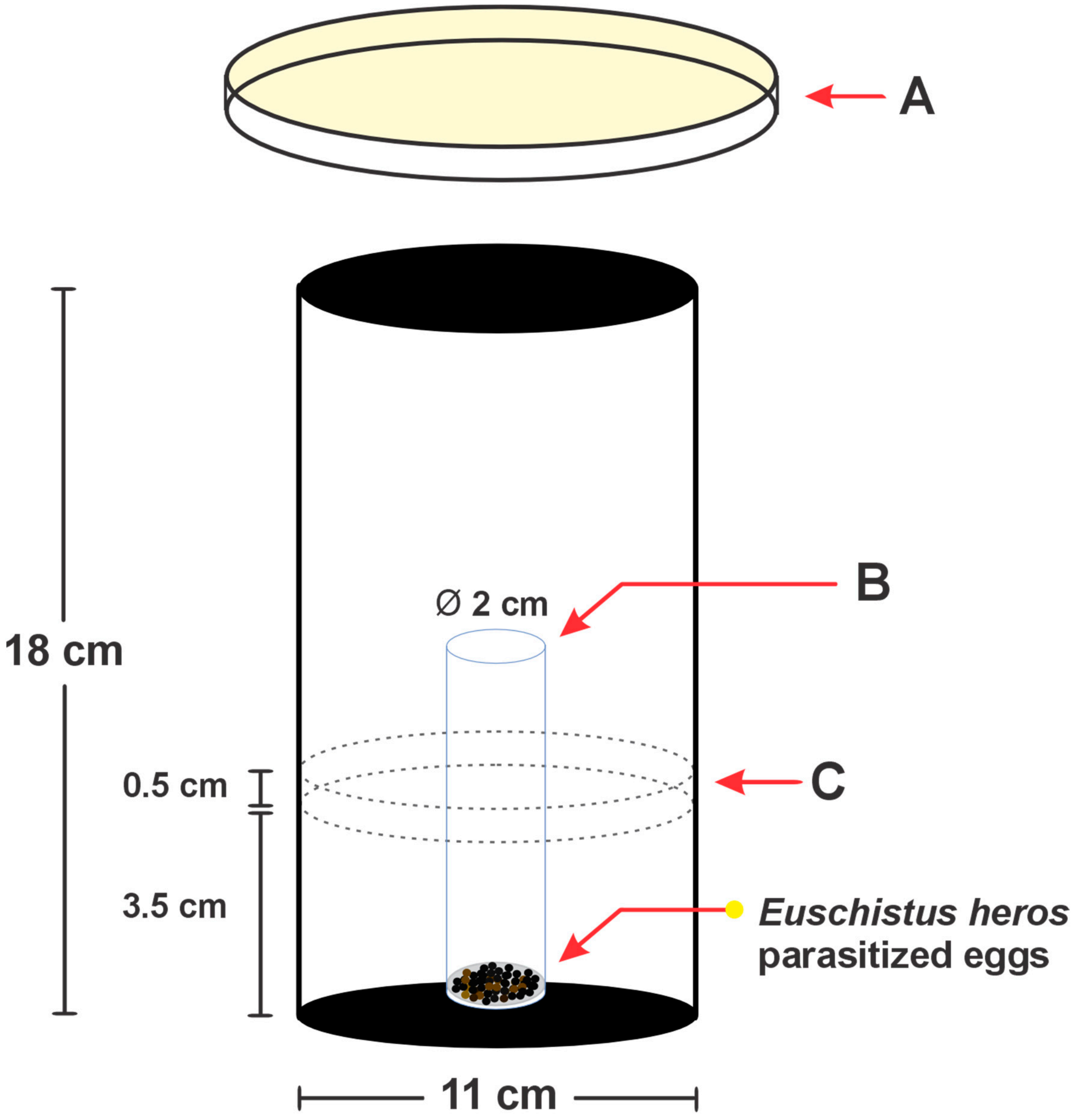

Figure S3 - Schematic illustration of the flight cage model recommended by the IOBC and adapted by Prezotti [10]: Petri dish coated with entomological glue for capturing flyers (A); flat-bottom glass tube containing the egg card from which *T. podisi* adults emerged and where immobile insects were observed (B); acetate ring positioned 3.5 cm from the base and coated with entomological glue for collecting walkers (C).
